# *ITPA* Genotypes Predict Anemia but Do Not Affect Virological Response with Interferon-Free Faldaprevir, Deleobuvir, and Ribavirin for HCV Infection

**DOI:** 10.1371/journal.pone.0144004

**Published:** 2015-12-09

**Authors:** Tarik Asselah, Stefan Zeuzem, Vicente Soriano, Jean-Pierre Bronowicki, Ansgar W. Lohse, Beat Müllhaupt, Marcus Schuchmann, Marc Bourlière, Maria Buti, Stuart K. Roberts, Edward J. Gane, Jerry O. Stern, Florian Voss, Patrick Baum, John-Paul Gallivan, Wulf O. Böcher, Federico J. Mensa

**Affiliations:** 1 Service d’Hépatologie, Hôpital Beaujon, INSERM UMR S 1149, CRI, Université Paris Diderot, DHU UNITY, Clichy, France; 2 J. W. Goethe University Hospital, Frankfurt, Germany; 3 Department of Infectious Diseases, Hospital Carlos III, Madrid, Spain; 4 INSERM U954, Centre Hospitalier Universitaire de Nancy, Université de Lorraine, Vandoeuvre les Nancy, France; 5 I. Medizinische Klinik und Poliklinik, University Hospital Hamburg-Eppendorf, Hamburg, Germany; 6 Division of Gastroenterology and Hepatology, University Hospital of Zurich, Zurich, Switzerland; 7 Department of Internal Medicine I, University Hospital Mainz, Mainz, Germany; 8 Service d’hépato-gastro-entérologie, Hôpital Saint Joseph, Marseille, France; 9 Liver Unit, Hospital Universitario Valle de Hebron and CIBERehd del Instituto Carlos III, Barcelona, Spain; 10 Department of Gastroenterology, Alfred Hospital, Melbourne, Australia; 11 Auckland Clinical Studies, Auckland, New Zealand; 12 Boehringer Ingelheim Pharmaceuticals, Inc., Ridgefield, CT, United States of America; 13 Boehringer Ingelheim Pharma GmbH & Co. KG, Ingelheim, Germany; 14 Boehringer Ingelheim Pharma GmbH & Co. KG, Biberach, Germany; Clínica Universidad de Navarra, SPAIN

## Abstract

**Background & Aim:**

Whether inosine triphosphatase (*ITPA*) gene polymorphisms predict anemia during interferon-free therapy in chronic hepatitis C virus (HCV)-infected patients is unknown. We examined the relationship between two *ITPA* polymorphisms, anemia, and sustained virological response 12 weeks post-treatment (SVR12) in patients receiving the NS3/4A protease inhibitor faldaprevir, the non-nucleoside polymerase inhibitor deleobuvir, and ribavirin.

**Methods:**

HCV genotype 1-infected, treatment-naïve patients (N = 362) were randomized and treated in one of five treatment arms with faldaprevir and deleobuvir with or without ribavirin. Two *ITPA* polymorphisms (rs1127354 and rs6051702) were genotyped and defined as ITPA-deficient (rs1127354 AA or AC; rs6051702 CC or CA) or ITPA-non-deficient (rs1127354 CC; rs6051702 AA) according to their association with ITPA deficiency. Baseline and on-treatment variables associated with anemia and SVR12 were identified using logistic regression.

**Results:**

In the pooled ribavirin-containing arms, 10.1% (32/316) of patients experienced on-treatment hemoglobin <10 g/dL, and 32.6% (103/316) experienced on-treatment hemoglobin <10 g/dL or a change from baseline ≥3.5 g/dL. Of the latter group, 99% (102/103) had the ITPA-non-deficient rs1127354 genotype. Other variables associated with on-treatment hemoglobin <10 g/dL or a decrease ≥3.5 g/dL were age, baseline hemoglobin, rs6051702 genotype, and plasma ribavirin concentration. In a multivariate analysis, high plasma ribavirin concentration, low baseline hemoglobin, HCV genotype 1b, and *IL28B* genotype CC were associated with higher SVR12.

**Conclusions:**

The *ITPA* rs1127354 CC and rs6051702 AA genotypes may predict ribavirin-induced anemia during treatment with interferon-free, ribavirin-containing regimens. With this interferon-free regimen, SVR was associated with ribavirin levels, but not with anemia or *ITPA* genotypes.

**Trial Registration:**

ClinicalTrials.gov: NCT01132313

## Introduction

Ribavirin is an important component of interferon-containing regimens for the treatment of chronic hepatitis C virus (HCV) infection, and is also included in many interferon-free regimens currently in development [1–5]. Among patients receiving ribavirin with peginterferon, anemia is common, often resulting in ribavirin dose reduction or discontinuation [1–3, 6]. Anemia is likely due to a ribavirin-associated, dose-dependent hemolytic anemia which may be worsened by the myelosuppressive action of peginterferon. Ribavirin-induced anemia has also been observed in trials of interferon-free, ribavirin-containing regimens in development for chronic HCV [4, 5, 7, 8]. For HCV genotype 2 infection, sofosbuvir plus ribavirin is recommended [9]. For HCV genotype 4 infection, sofosbuvir plus ribavirin for 24 weeks has been proposed [10]. The interferon-free paritaprevir (previously ABT-450)-based regimen also contains ribavirin [5]. Predicting anemia during treatment with ribavirin-containing, interferon-free regimens is essential for optimal patient management, particularly when treating special populations such as patients with high cardiovascular risk.

Genetic variation in the inosine triphosphatase (*ITPA*) gene on chromosome 20 is associated with the development of anemia during peginterferon and ribavirin therapy [11–13]. Single-nucleotide polymorphisms (SNPs) at or near this locus have been found to be associated with ribavirin-induced anemia. The mechanism underlying this association is not well understood, but one hypothesis is that reduced ITPA activity in erythrocytes leads to higher levels of inosine triphosphate (ITP). ITP, in turn, can substitute for guanosine triphosphate, allowing the synthesis of adenosine triphosphate (ATP), and the resulting protection against ATP depletion may prevent ribavirin-induced anemia [14, 15]. For the clinician, determining the presence of *ITPA* SNPs may help predict the development of anemia during chronic HCV treatment. There are currently no reports in the peer-reviewed literature on the effects of *ITPA* SNPs on ribavirin-induced anemia during interferon-free therapy.

The SOUND-C2 study was a phase 2b, randomized, open-label study of the HCV NS3/4A protease inhibitor faldaprevir and the non-nucleoside NS5B polymerase inhibitor deleobuvir with or without ribavirin in treatment-naïve, chronic HCV genotype 1-infected patients [16]. Efficacy results showed that rates of sustained virological response at 12 weeks post-treatment (SVR12) were significantly higher in the ribavirin-containing arms (52–69%) than in the ribavirin-free arm (39%; *p* = 0.003) [16]. Rates were higher among genotype 1b-infected patients (up to 85% and 57% in the ribavirin-containing and ribavirin-free arms, respectively), and among patients who completed therapy (i.e., excluding those who discontinued early for reasons unrelated to efficacy; 66–72% and 46% in the ribavirin-containing and ribavirin-free arms, respectively) [16], (BI data on file). We analysed hemoglobin reductions in patients treated with faldaprevir, deleobuvir, and ribavirin in the SOUND-C2 study. This report describes the incidence of anemia during SOUND-C2 and any baseline factors associated with anemia. We specifically analysed two SNPs, one within (rs1127354) and one adjacent to (rs6051702) the *ITPA* gene.

Since the completion of this analysis, the development of faldaprevir and deleobuvir has been terminated. However, since ribavirin is an important component of many interferon-free regimens, and the effects of *ITPA* polymorphisms on interferon-free, ribavirin-containing regimens are unknown, this analysis provides information that may be relevant to other HCV treatment regimens.

## Materials and Methods

### Study design and patient population

In the SOUND-C2 study, 362 HCV genotype 1-infected, treatment-naïve patients were randomized and treated in one of five treatment groups: faldaprevir 120 mg once daily and deleobuvir 600 mg three times daily plus ribavirin for 16 weeks (TID16W), 28 weeks (TID28W), or 40 weeks (TID40W); faldaprevir 120 mg once daily and deleobuvir 600 mg twice daily plus ribavirin for 28 weeks (BID28W); or faldaprevir 120 mg once daily and deleobuvir 600 mg three times daily, without ribavirin, for 28 weeks (TID28W-NR) [16]. Randomization was stratified by viral subtype (1a or 1b) and by *IL28B* (rs12979860) genotype (CC or non-CC). The primary efficacy end point was SVR12 (undetectable HCV RNA at 12 weeks post-treatment). Ribavirin was dosed twice daily at 1000–1200 mg per day according to body weight (1000 mg for body weight <75 kg; 1200 mg for body weight ≥75 kg). Ribavirin dose reductions and erythropoietin use were permitted for the management of anemia. Hemoglobin levels were measured at baseline, day 4, weeks 1, 2, 4, 6, and 8, then every 4 weeks through the end of treatment.

The study was approved by the institutional review board/independent ethics committee of each participating site, and was carried out in compliance with the ethical guidelines of the Declaration of Helsinki and in accordance with the International Conference on Harmonisation Guidelines for Good Clinical Practice. All patients provided written informed consent prior to enrolment, and gave separate consent for genetic testing.

### Statistical analysis

Anemia was defined as on-treatment hemoglobin <10 g/dL or a decrease from baseline of ≥3.5 g/dL, consistent with Division of AIDS Table for Grading the Severity of Adult and Pediatric Adverse Events grade ≥2 changes. Categorical variables are reported as frequencies and percentages. Continuous data are expressed as means. Using pooled data from the ribavirin-containing arms, baseline and on-treatment variables significantly (determined by *p*<0.05) associated with the development of anemia were identified using logistic regression. The analysis was restricted to patients with the ITPA-non-deficient rs1127354 CC genotype because nearly all patients with anemia had the ITPA-non-deficient CC genotype at this locus, and only one patient with an ITPA-deficient rs1127354 genotype had anemia, consistent with what has been reported in the literature [11, 12]. Variables identified as significant in the univariate analysis were evaluated using multivariate logistic regression. Using the data set of patients who completed therapy (excluding those who discontinued for reasons unrelated to efficacy), univariate and multivariate logistic regression analyses were used to identify baseline and on-treatment factors associated with SVR12.

### *ITPA* genotyping

Genomic DNA was extracted using magnetic bead technology. DNA concentration and quality was determined by absorbance measurements at 260 nm and 280 nm using a μQuant^™^ microplate spectrophotometer. *ITPA* genotyping (rs1127354 and rs6051702) was performed by high resolution melting curve analysis using a LightCycler^®^ 480 real-time PCR system. *ITPA* genotypes were defined as ITPA-deficient (rs1127354 AA or AC and rs6051702 CC or CA) or ITPA-non-deficient (rs1127354 CC and rs6051702 AA) based on their association with ITPA deficiency and hemolytic anemia in previous studies [11, 12]. The distribution of genotypes was tested by Hardy-Weinberg equilibrium.

## Results

### Patient characteristics and incidence of anemia

Baseline demographic and disease characteristics are shown in Table 1. The majority of patients were white and approximately half were male. The mean age of patients (45.3–48.9 years) and baseline hemoglobin levels (14.8–15.1 g/dL) were similar across the arms. Nearly one-quarter of patients (23%; 85/362) had advanced fibrosis (F3 or F4). The highest rates of cirrhosis were in the TID16W (11.1%) and BID28W (11.5%) arms.

**Table 1:** Baseline demographics and disease characteristics.

Parameter	TID16W (N = 81)	TID28W (N = 80)	TID40W (N = 77)	BID28W (N = 78)	TID28W-NR (N = 46)
**Male, n (%)**	45 (55.6)	41 (51.3)	36 (46.8)	41 (52.6)	24 (52.2)
**Race, n (%)**					
White	79 (97.5)	78 (97.5)	76 (98.7)	77 (98.7)	46 (100.0)
Black/African American	2 (2.5)	1 (1.3)	0	1 (1.3)	0
Asian	0	1 (1.3)	0	0	0
Other	0	0	1 (1.3)	0	0
**Age, mean (SD), years**	48.6 (11.3)	47.3 (11.2)	48.9 (10.7)	47.9 (11.1)	45.3 (13.0)
**Body mass index, mean kg/m^2^**	25.3	25.5	24.8	25.0	25.5
**Cirrhosis, n (%)**	9 (11.1)	7 (8.8)	5 (6.5)	9 (11.5)	3 (6.5)
***IL28B* genotype, n (%)**					
CC	21 (25.9)	21 (26.3)	19 (24.7)	19 (24.4)	12 (26.1)
Non-CC	60 (74.1)	58 (72.5)	58 (75.3)	59 (75.6)	33 (71.7)
Missing	0	1 (1.3)	0	0	1 (2.2)
**Genotype 1 subtype, n (%)**					
1a	34 (42.0)	32 (40.0)	34 (44.2)	30 (38.5)	18 (39.1)
1b	47 (58.0)	48 (60.0)	43 (55.8)	48 (61.5)	28 (60.9)
**HCV RNA ≥800,000 IU/mL, n (%)**	70 (86.4)	66 (82.5)	67 (87.0)	66 (84.6)	36 (78.3)
**Baseline hemoglobin, mean (SD), g/dL**	15.1 (1.0)	14.8 (1.0)	14.9 (1.0)	14.8 (1.3)	15.0 (1.0)
**Fibrosis stage,** ^a^ **n (%)**					
≤F2	63 (77.8)	58 (72.5)	62 (80.5)	57 (73.1)	35 (76.1)
F3–F4	17 (21.0)	21 (26.3)	15 (19.5)	21 (26.9)	11 (23.9)
Missing	1 (1.2)	1 (1.3)	0	0	0

^a^Fibrosis stage was determined by either METAVIR score or fibroscan result; if a METAVIR score was not available, then a fibroscan result was used (fibroscan <9.5 = ≤F2 and fibroscan ≥9.5 = ≥F3). TID16W/TID28W/TID40W, faldaprevir 120 mg once daily and deleobuvir 600 mg three times daily plus ribavirin for 16, 28, and 40 weeks, respectively; BID28W, faldaprevir 120 mg once daily and deleobuvir 600 mg twice daily plus ribavirin for 28 weeks; TID28W-NR, faldaprevir 120 mg once daily and deleobuvir 600 mg three times daily, without ribavirin, for 28 weeks.

**Fig 1:**
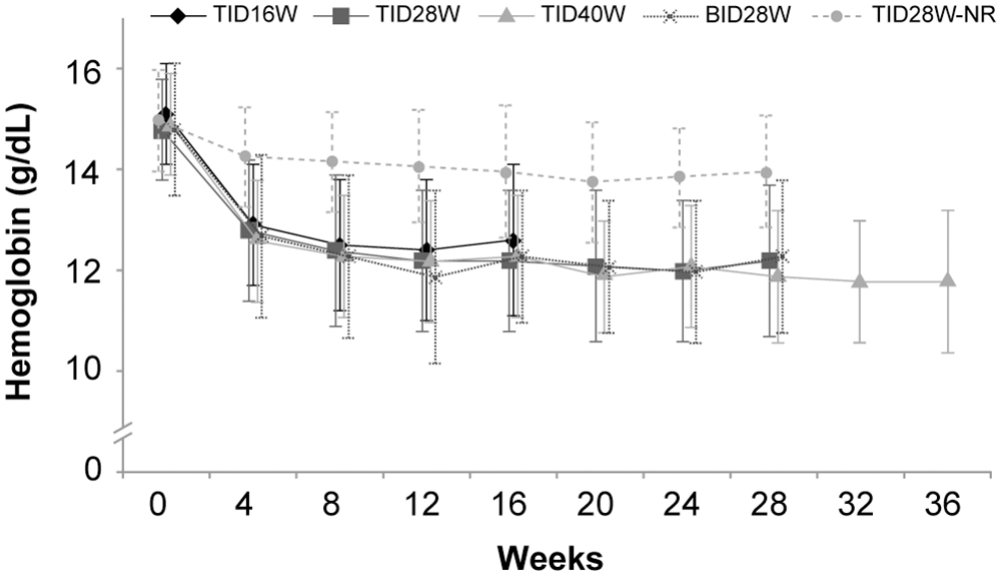
Hemoglobin levels over time. Mean hemoglobin by treatment group over time is shown. The mean reduction at the end of treatment for the pooled ribavirin-containing arms was approximately 2.5 g/dL. TID16W/TID28W/TID40W, faldaprevir 120 mg once daily, deleobuvir 600 mg three times daily, and ribavirin for 16, 28, and 40 weeks, respectively; BID28W, faldaprevir 120 mg once daily, deleobuvir 600 mg twice daily, and ribavirin for 28 weeks; TID28W-NR, faldaprevir 120 mg once daily and deleobuvir 600 mg three times daily without ribavirin for 28 weeks.

**Fig 2:**
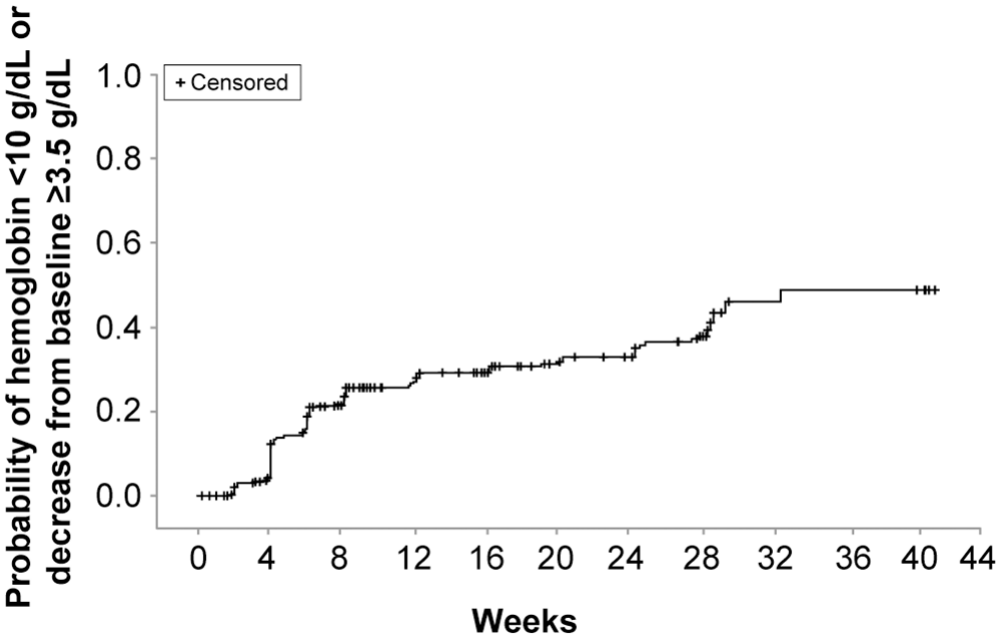
Probability of anemia over time. Among patients in the pooled ribavirin-containing arms with anemia, the median time to the first occurrence of anemia was 42 days.

Anemia was not detected among patients in the ribavirin-free arm [16]; therefore, this analysis focuses principally on patients in the ribavirin-containing arms. Of 316 patients who received ribavirin, 103 (32.6%) had on-treatment hemoglobin <10 g/dL (32 patients, 10.1%) or a change from baseline of ≥3.5 g/dL. The mean reduction in hemoglobin at the end of treatment in the pooled ribavirin-containing arms was approximately 2.5 g/dL. Fig 1 shows mean hemoglobin levels by treatment group over time. Among patients in the ribavirin-containing arms with an on-treatment hemoglobin of <10 g/dL or a change from baseline of ≥3.5 g/dL, the median time to the first occurrence of such an event was 42 days. Fig 2 shows the probability of anemia (hemoglobin <10 g/dL or decrease of ≥3.5 g/dL) over time for the pooled ribavirin-containing arms.

Reductions in hemoglobin levels appear not to have had an impact on SVR12. As shown in Table 2, 65% (67/103) of patients who had hemoglobin <10 g/dL or a decrease from baseline ≥3.5 g/dL achieved SVR12, compared with 57% (122/213) of patients who did not experience anemia.

**Table 2:** Rates of SVR12 by incidence of anemia in pooled ribavirin-containing arms.

n/N (%)	TID16W	TID28W	TID40W	BID28W	Total
**All patients, Hb ≥10 g/dL and no reduction ≥3.5 g/dL**	33/57 (58)	32/55 (58)	22/49 (45)	35/52 (67)	122/213 (57)
Genotype 1a	10/26 (38)	10/24 (42)	10/24 (42)	8/20 (40)	38/94 (40)
Genotype 1b	23/31 (74)	22/31 (71)	12/25 (48)	27/32 (84)	84/119 (71)
**All patients, Hb <10 g/dL or reduction ≥3.5 g/dL**	15/24 (63)	15/25 (60)	18/28 (64)	19/26 (73)	67/103 (65)
Genotype 1a	3/8 (38)	4/8 (50)	6/10 (60)	5/10 (50)	18/36 (50)
Genotype 1b	12/16 (75)	11/17 (65)	12/18 (67)	14/16 (88)	49/67 (73)

Hb, hemoglobin; SVR12, sustained virological response (undetectable HCV RNA) at 12 weeks post-treatment; TID16W/TID28W/TID40W, faldaprevir 120 mg once daily and deleobuvir 600 mg three times daily plus ribavirin for 16, 28, and 40 weeks, respectively; BID28W, faldaprevir 120 mg once daily and deleobuvir 600 mg twice daily plus ribavirin for 28 weeks.

### *ITPA* SNPs and anemia events

**Fig 3:**
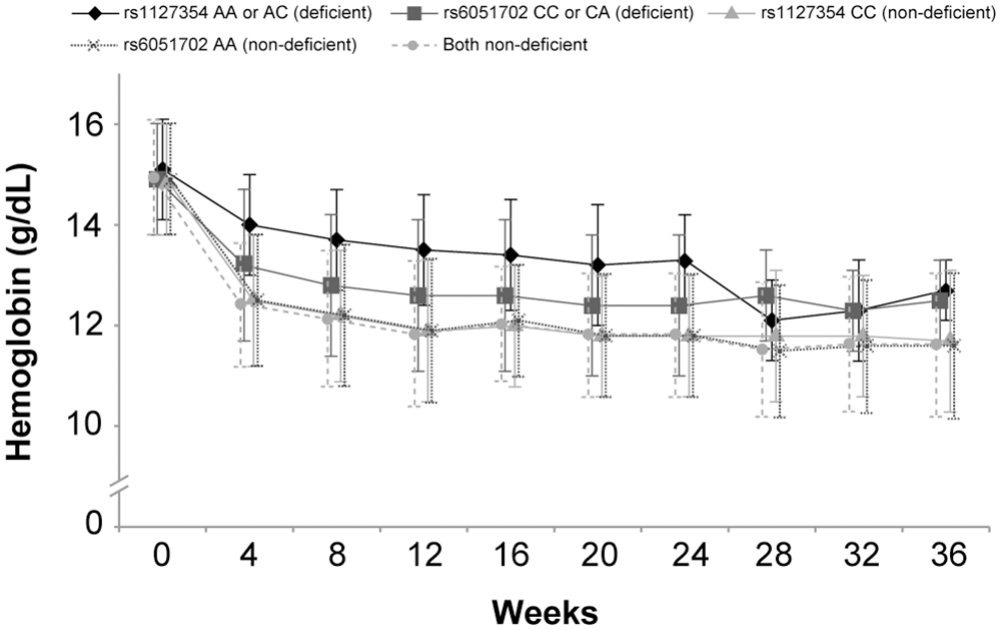
Mean hemoglobin levels by inosine triphosphatase (*ITPA*) gene single-nucleotide polymorphisms, pooled ribavirin-containing arms.

*ITPA* data were available for 360 of the 362 patients in the study. Genotype frequency for each variant was in agreement with Hardy–Weinberg equilibrium in the tested population. The vast majority of patients (81–91%) across all arms had the ITPA-non-deficient rs1127354 CC genotype, while 58–76% of patients had the ITPA-non-deficient rs6051702 AA genotype (Table 3). Almost all patients with the rs6051702 AA genotype had the ITPA-non-deficient genotype at both positions (rs6051702 and rs1127354). Patients with the rs1127354 CC genotype were observed to have a greater reduction in mean hemoglobin over time than those with ITPA-deficient genotypes (AA/AC) at this position (Fig 3). The difference between mean hemoglobin levels over time was less pronounced between those with ITPA-deficient and–non-deficient rs6051702 genotypes (Fig 3).

**Table 3:** Inosine triphosphatase (*ITPA*) gene single-nucleotide polymorphisms by treatment arm.

	TID16W	TID28W	TID40W	BID28W	TID28W-NR
**Patients with *ITPA* data, N**	80	80	77	77	46
**rs1127354, n (%)**					
ITPA-deficient (AA/AC)	10 (13)	9 (11)	11 (14)	15 (19)	4 (9)
ITPA-non-deficient (CC)	70 (88)	71 (89)	66 (86)	62 (81)	42 (91)
**rs6051702, n (%)**					
ITPA-deficient (CC/CA)	28 (35)	34 (43)	25 (32)	25 (32)	11 (24)
ITPA-non-deficient (AA)	52 (65)	46 (58)	52 (68)	52 (68)	35 (76)
**ITPA-non-deficient genotype at both positions, n (%)**					
No	31 (39)	35 (44)	28 (36)	31 (40)	11 (24)
Yes	49 (61)	45 (56)	49 (64)	46 (60)	35 (76)

TID16W/TID28W/TID40W, faldaprevir 120 mg once daily and deleobuvir 600 mg three times daily plus ribavirin for 16, 28, and 40 weeks, respectively; BID28W, faldaprevir 120 mg once daily and deleobuvir 600 mg twice daily plus ribavirin for 28 weeks; TID28W-NR, faldaprevir 120 mg once daily and deleobuvir 600 mg three times daily, without ribavirin, for 28 weeks.

### Predictors of anemia and virological response

Only one of the 103 patients with anemia (hemoglobin <10 g/dL or decreased from baseline by ≥3.5 g/dL) had the ITPA-deficient AA or AC genotype at rs1127354. Ninety-nine percent (102/103) had the ITPA-non-deficient rs1127354 CC genotype, while 77% (79/103) had the ITPA-non-deficient rs6051702 AA genotype (Table 4). Therefore, the probability of experiencing an anemia event (hemoglobin <10 g/dL or decreased by ≥3.5 g/dL) based on selected baseline and on-treatment factors was explored among patients with the rs1127354 CC genotype only. Using logistic regression analysis on pooled data from the ribavirin-containing treatment arms, the association of anemia with the following factors was explored: gender, age (<60 years vs ≥60 years), weight (<75 kg vs ≥75 kg), body mass index (<25 kg/m^2^ vs ≥25 kg/m^2^), fibrosis stage (F0–F2 vs F3–F4), presence of cirrhosis (yes/no), baseline hemoglobin (<15 g/dL vs ≥15 g/dL), rs6051702 genotype (ITPA-deficient (CC/CA) vs ITPA-non-deficient (AA])), initial ribavirin dose, and faldaprevir, deleobuvir, and ribavirin trough concentrations through week 12. Factors found to be significantly associated with hemoglobin <10 g/dL or decreased by ≥3.5 g/dL in a univariate analysis were age, weight, baseline hemoglobin, rs6051702 genotype, and plasma ribavirin concentration (Table 5). With the exception of weight, these factors remained significant in a multivariate analysis. Plasma levels of faldaprevir and deleobuvir were not associated with anemia.

**Table 4:** Effect of *ITPA* SNPs on hemoglobin level, ribavirin dose, and erythropoietin use, pooled ribavirin-containing arms.

*ITPA* SNP and genotype, n (%)	rs1127354 ITPA-deficient AA or AC (n = 45)	rs1127354 ITPA-non-deficient CC (n = 269)	rs6051702 ITPA-deficient CC or CA (n = 112)	rs6051702 ITPA-non-deficient AA (n = 202)	ITPA-non-deficient genotype at both positions (n = 189)
Hb <10 g/dL or decrease from baseline ≥3.5 g/dL	1 (2.2)	102 (37.9)	24 (21.4)	79 (39.1)	79 (41.8)
Ribavirin dose reduction for anemia^a^	1 (2.2)	17 (6.3)	1 (0.9)	17 (8.4)	16 (8.5)
Erythropoietin use	0	13 (4.8)	2 (1.8)	11 (5.4)	11 (5.8)

^a^Anemia as an adverse event defined by investigators (not a laboratory event).

*ITPA*, inosine triphosphatase gene; SNP, single nucleotide polymorphism; Hb, hemoglobin.

**Table 5:** Factors associated with anemia in univariate and multivariate analyses, all treated patients.

Factor	Odds Ratio	95% CI	*p*-value
**Univariate analysis**			
Log2 (median trough ribavirin through week 12)	5.35	2.86, 9.98	<0.0001
Baseline hemoglobin, g/dL, <15 vs ≥15	0.38	0.23, 0.64	0.0002
Age, years, <60 vs ≥60	0.33	0.16, 0.65	0.0014
Weight, <75 kg vs ≥75 kg	0.61	0.37, 1.00	0.0495
rs6051702, ITPA-deficient (CC/CA) vs–non-deficient (AA)	0.56	0.32, 0.99	0.0451
**Multivariate analysis**			
Log2 (median trough ribavirin through week 12)	10.62	4.70, 23.99	<0.0001
Baseline hemoglobin, g/dL, <15 vs ≥15	0.14	0.07, 0.28	<0.0001
Age, years, <60 vs ≥60	0.32	0.13, 0.80	0.0147
rs6051702, ITPA-deficient (CC/CA) vs–non-deficient (AA)	0.44	0.22, 0.86	0.0163

Ribavirin-containing arms are pooled and all patients have the rs1127354 CC genotype. Covariates included age (<60 years vs ≥60 years), gender, weight (<75 kg vs ≥75 kg), fibrosis stage (F0–F2 vs F3–F4), presence of cirrhosis (yes/no), body mass index (<25 kg/m^2^ vs ≥25 kg/m^2^), rs6051702 genotype (ITPA-deficient (CC/CA) vs–non-deficient (AA)), baseline hemoglobin (<15 g/dL vs ≥15 g/dL), faldaprevir, deleobuvir, and ribavirin trough levels through week 12, and baseline ribavirin dose. ITPA, inosine triphosphatase.

Since virological response has been reported to be associated with ribavirin dosing or anemia, we also explored the association between SVR12 and baseline and on-treatment factors using the data set of patients who completed therapy (excluding those who discontinued for reasons unrelated to efficacy). Covariates included initial ribavirin dose, ribavirin median trough plasma concentration through week 12 (measured at every scheduled visit), baseline hemoglobin, incident anemia (hemoglobin <10 g/dL or change of ≥3.5 g/dL), treatment group, and rs1127354 and rs6051702 genotypes, in addition to factors known to be associated with SVR12 (*IL28B* genotype, genotype 1 subtype, baseline HCV RNA, baseline gamma glutamyl transferase (GGT), fibrosis stage, presence of cirrhosis, gender, and age). In a univariate analysis using pooled data from the ribavirin-containing arms, we found that ribavirin concentrations through week 12, baseline hemoglobin, and baseline ribavirin dose were significantly associated with SVR12, as were the more established predictors including age, genotype 1 subtype, *IL28B* genotype, GGT, gender, and baseline viral load (Table 6). Neither rs1127354 nor rs6051702 genotype was found to be associated with SVR12. In a multivariate analysis, ribavirin concentrations, baseline hemoglobin, genotype 1 subtype, and *IL28B* genotype remained significantly associated with SVR12 (Table 6).

**Table 6:** Covariates evaluated for association with SVR12 in univariate and multivariate analyses.

Factor	Odds Ratio	95% CI	*p*-value
**Univariate analysis**			
Log2 (median trough ribavirin through week 12)	7.37	3.71, 14.64	<0.0001
Baseline hemoglobin, g/dL, <15 vs ≥15	3.75	2.15, 6.56	<0.0001
Age, years, <60 vs ≥60	0.11	0.03, 0.49	0.0034
Baseline ribavirin dose per day and kg	1.16	1.01, 1.33	0.0305
Genotype 1 subtype, 1b vs 1a	6.04	3.43, 10.63	<0.0001
*IL28B* genotype, CC vs non-CC	2.85	1.41, 5.78	0.0036
Baseline GGT, elevated vs normal	0.41	0.25, 0.70	0.0010
Gender, male vs female	0.33	0.19, 0.58	<0.0001
Baseline HCV RNA, ≥800,000 IU/mL vs <800,000 IU/mL	0.39	0.16, 0.98	0.0457
rs1127354, ITPA-deficient (AA/AC) vs–non-deficient (CC)	1.00	0.49, 2.03	0.9915
rs6051702, ITPA-deficient (CC/CA) vs–non-deficient (AA)	1.31	0.76, 2.26	0.3303
Hb <10 g/dL or change from baseline ≥3.5 g/dL, yes vs no	1.25	0.72, 2.16	0.43222
Fibrosis stage, F0–F2 vs F3–F4	0.76	0.40, 1.41	0.3823
Cirrhosis, yes vs no	0.95	0.39, 2.30	0.9110
Randomized treatment group			
TID16W vs BID28W	0.75	0.37, 1.50	0.4123
TID28W vs BID28W	0.87	0.42, 1.79	0.7056
TID40W vs BID28W	0.86	0.41, 1.83	0.7032
**Multivariate analysis**			
Log2 (median trough ribavirin through week 12)	7.86	3.31, 18.67	<0.0001
Baseline hemoglobin, g/dL, <15 vs ≥15	3.35	1.71, 6.59	0.0004
Genotype 1 subtype, 1b vs 1a	6.01	3.06, 11.79	<0.0001
*IL28B* genotype, CC vs non-CC	7.05	2.90, 17.12	<0.0001

Based on patients who completed therapy (excluding those who discontinued for reasons unrelated to efficacy). Ribavirin-containing arms are pooled. *ITPA*, inosine triphosphatase; GGT, gamma glutamyl transferase; Hb, hemoglobin; TID16W, faldaprevir 120 mg once daily, deleobuvir 600 mg three times daily, and ribavirin for 16 weeks; BID28W, faldaprevir 120 mg once daily, deleobuvir 600 mg twice daily, and ribavirin for 28 weeks; TID40W, faldaprevir 120 mg once daily, deleobuvir 600 mg three times daily, and ribavirin for 40 weeks.

Ribavirin dose reductions for anemia (as defined by the investigator, not specified by laboratory parameters) were more frequent among patients with the ITPA-non-deficient rs1127354 CC genotype than among those with the AA or AC genotype (6.3% vs 2.2%; Table 4), and were similarly more frequent among those with the ITPA-non-deficient rs6051702 AA genotype than among those with the CC or CA genotype (8.4% vs 0.9%). Across all ribavirin-containing treatment groups, SVR12 rates were lower among patients who had ribavirin dose reductions or interruptions than among those who did not (TID16W, 40% (2/5) vs 61% (46/76); TID28W, 25% (3/12) vs 65% (44/68); TID40W, 42% (5/12) vs 54% (35/65); and BID28W, 40% (4/10) vs 74% (50/68)).

## Discussion

The results of this study suggest that *ITPA* SNPs may help predict anemia in chronic hepatitis C patients treated with interferon-free regimens that contain ribavirin. We found that the ITPA-non-deficient genotypes rs1127354 CC and rs6051702 AA were associated with the development of anemia during treatment with faldaprevir, deleobuvir, and ribavirin in the SOUND-C2 study. Ribavirin is an important component of several interferon-free regimens [5, 8], and studies have shown the benefit of including ribavirin with direct-acting antivirals, for example, in patients treated with the combination of paritaprevir/ritonavir/ombitasvir and dasabuvir who are infected with HCV genotype 1a, HCV genotype 1b in the presence of cirrhosis, or HCV genotype 4 [5, 17, 18, 19]. Most interferon-free regimens recommended for patients with cirrhosis contain ribavirin [9]. Although the development of faldaprevir and deleobuvir has been halted, our findings remain relevant since they may apply to treatment with other interferon-free, ribavirin-containing regimens.

In the SOUND-C2 study, 10% (32/316) of patients in the pooled ribavirin-containing arms had a hemoglobin level <10 g/dL. This incidence of hemoglobin <10 g/dL is considerably lower than that observed during studies of peginterferon-containing regimens. In the STARTVerso1 study, 26% of patients receiving faldaprevir with peginterferon and ribavirin had a hemoglobin level <10 g/dL, as did 30% of patients receiving peginterferon and ribavirin in the IDEAL study, and 50% of those receiving boceprevir plus peginterferon and ribavirin in the SPRINT-2 study [1, 20, 21]. The higher rates of anemia in these studies suggest that the myelosuppressive action of peginterferon may contribute to hemoglobin reduction during chronic HCV therapy, although caution should be exercised when comparing data across studies.

A small increase in the SVR12 rate was observed among patients who developed hemoglobin <10 g/dL or a decrease from baseline of ≥3.5 g/dL, compared with patients who did not have such hemoglobin reductions (65% vs 57%). This observed difference in SVR12 may be due in part to differential rates of early discontinuation, since patients who discontinue early are both less likely to experience anemia and less likely to achieve SVR12 than those who continue treatment. Higher SVR rates among patients with incident anemia have been observed during treatment with peginterferon-containing regimens [1, 20]. However, it has been suggested that anemia is only a marker for ribavirin exposure and that higher SVR rates in anemic patients may be due to higher plasma ribavirin levels [20, 22, 23].

Our analysis of *ITPA* SNPs showed that patients with the ITPA-non-deficient rs112354CC genotype had greater hemoglobin reductions over time than patients with the ITPA-deficient AA or AC genotype. Of 103 patients with hemoglobin <10 g/dL or a decrease of ≥3.5 g/dL, all but one had the ITPA-non-deficient rs112735CC genotype. Our finding of a strong association between *ITPA* genotypes and the development of anemia is consistent with the results of earlier studies. Fellay and co-workers found that among patients receiving peginterferon and ribavirin, SNPs at positions rs6051702 and rs1127354 were independently associated with the development of anemia [11], while others have shown that *ITPA* genotypes are associated with anemia during triple therapy including protease inhibitors [24, 25]. Consistent with those earlier studies in patients receiving peginterferon-containing regimens, our results show that the *ITPA* rs1127354 CC genotype is a predictor of ribavirin-induced anemia during interferon-free therapy.

Among patients with the rs1127354 CC genotype, we found that other independent predictors of anemia were age ≥60 years, baseline hemoglobin ≥15 g/dL, the rs6051702 AA genotype, and plasma ribavirin levels. Others have found that low baseline hemoglobin is a predictor of anemia during treatment with telaprevir-based triple therapy [25]. Our finding that a high baseline hemoglobin is associated with anemia is likely due to the definition of anemia in this analysis—hemoglobin <10 g/dL or decreased from baseline by ≥3.5 g/dL; patients with high baseline hemoglobin appear to be more likely than those with lower levels to experience a decrease of ≥3.5 g/dL. The predictors of anemia that we identified in the context of interferon-free treatment can help clinicians identify patients who may need to be monitored more closely during ribavirin-containing, interferon-free therapy, including patients with advanced age, low baseline hemoglobin, or with the ITPA-non-deficient genotypes at both the rs1127354 and the rs6051702 positions. Early dose reduction of ribavirin or use of erythropoietin may help manage anemia in susceptible patients.

Since treatment response has been reported to be associated with ribavirin dose and/or anemia, we also explored predictors of SVR12, taking into account *ITPA* genotypes at both the rs1127354 and the rs6051702 positions, baseline hemoglobin levels, and on-treatment ribavirin concentrations. Multivariate analysis showed that ribavirin concentration, baseline hemoglobin, genotype 1 subtype, and *IL28B* genotype were independent predictors of SVR12. Neither *ITPA* SNP was associated with SVR12. This may seem surprising since we found a strong association between both ITPA-non-deficient genotypes and anemia. However, as some researchers have suggested, anemia may not be directly related to SVR: Holmes and co-workers recently showed in a large cohort of peginterferon- and ribavirin-treated patients that the relationship between anemia and SVR is not mechanistic, and is most likely explained by plasma ribavirin levels [23]. Our results support this theory: in our study, plasma ribavirin concentrations were independently associated with SVR12 (based on the multivariate analysis) while the impact of ribavirin-induced anemia on SVR12 was minimal. This may reflect the fact that effective levels of ribavirin were also achieved in patients without anemia, who were most likely protected by ITPA-deficient genotypes. The inclusion of plasma ribavirin levels in the multivariate analysis enabled us to show that ribavirin exposure, and not anemia, is a predictor of SVR12.

Our understanding of the role of host genetics in HCV treatment response and tolerability is evolving, and will help clinicians tailor treatments to maximize response and minimize adverse events. Host *IL28B* genotype is known to be a strong predictor of response to interferon-based therapy [26, 27], while patients with variants in the *ITPA* gene are known to be protected against ribavirin-induced anemia during treatment with peginterferon-containing regimens [12]. The standard of care for chronic hepatitis C now includes interferon-free regimens, some including ribavirin and others ribavirin-free [5, 8, 28]. Our results suggest that *ITPA* genotypes can predict anemia during treatment with interferon-free, ribavirin-containing regimens. We also showed that in patients receiving such a regimen, SVR is not associated with anemia, and is associated with plasma ribavirin levels. One implication of our study is that patients known to have a genotype that does not protect against anemia may require more monitoring during treatment with ribavirin-containing, interferon-free regimens. Future identification of additional SNPs associated with anemia or with other adverse effects will help optimize therapy by identifying the best treatment regimens and management strategies for individual patients.
